# Functional and Structural Characterization of LRRK2 p.V1447L in Parkinson's Disease

**DOI:** 10.1002/mds.30284

**Published:** 2025-07-30

**Authors:** Neringa Pratuseviciute, Pawel Lis, Sacha Weber, Nicolas Gruchy, Lionel Arnaud, Dario R. Alessi, Esther Sammler

**Affiliations:** ^1^ Medical Research Council Protein Phosphorylation and Ubiquitylation Unit University of Dundee Dundee UK; ^2^ Institut du Cerveau‐Paris Brain Institute ICM Sorbonne Université, Inserm 1127, CNRS 7225, Hôpital de la Pitié Salpêtrière Paris France; ^3^ Université Caen Normandie, Normandie Université, Biotargen UR7450, CHU de Caen, Service de Génétique Caen France; ^4^ Département de Génétique Médicale APHP Sorbonne Université Paris France; ^5^ Division of Neuroscience, School of Medicine University of Dundee Dundee UK

**Keywords:** LRRK2, Parkinson's disease, genetics, peripheral blood neutrophils, Rab10 phosphorylation

## Abstract

**Background:**

Gain‐of‐kinase‐function variants in LRRK2 are a leading cause of monogenic Parkinson's disease (PD).

**Objectives:**

We tested the functional impact of a novel LRRK2 variant p.V1447L identified in a young‐onset PD patient in vivo in peripheral blood, as well as in a robust cellular assay, alongside other variants in close proximity to V1447.

**Methods:**

We measured LRRK2‐dependent Rab10 phosphorylation in neutrophils and monocytes of a LRRK2 p.V1447L carrier with PD. We performed structural mapping and evaluated the potential impact of other LRRK2 variants at and around LRRK2 V1447.

**Results:**

LRRK2 p.V1447L strongly increases LRRK2 kinase activity. We identified additional variants in the LRRK2 ROC:COR_B_ interface with critical impact on kinase activity and demonstrated that different substitutions at the same residue can have opposing effects.

**Conclusions:**

We recommend reclassifying LRRK2 p.V1447L from variant of uncertain significance to likely pathogenic. Our study expands the range of putative loss‐of‐kinase function variants to LRRK2 missense variants. © 2025 The Author(s). *Movement Disorders* published by Wiley Periodicals LLC on behalf of International Parkinson and Movement Disorder Society.

The global increase in the number of people living with Parkinson's disease (PD), now over 10 million cases, highlights the urgent need for disease‐modifying treatments.^1^ Clinical trials targeting molecular pathways linked to PD, particularly those involving genetic factors, are therefore of great interest. The leucine‐rich repeat kinase 2 (LRRK2) is a high‐value target for disease modification in PD with clinical trials currently underway.^2^, ^3^ Heterozygous gain‐of‐kinase function variants in *LRRK2* are the most common cause of monogenetic PD, accounting for 1%–4% of all PD cases, though this estimate is based primarily on common variants such as p.G2019S and p.R1441G.^4^, ^5^, ^6^ However, many rare LRRK2 variants of uncertain significance (VUS) remain uncharacterized, highlighting the need to define their functional impact and pathogenicity.^7^, ^8^ This has implications for patient stratification, genetic counseling, and potential eligibility for LRRK2‐targeted therapeutics.

Here, we report on the functional analysis of a novel LRRK2 VUS (p.V1447L) identified in a sporadic, early‐onset PD patient. The V1447 residue is located within a region of the GTPase domain of LRRK2 known to play an important role in controlling kinase activity. We show that V1447L greatly increases LRRK2 kinase activity in vivo in patient peripheral blood and in a robust cellular LRRK2 overexpression assay. Structural mapping suggests that V1447L might disrupt a critical regulatory interface promoting kinase activation by destabilizing the inactive conformation of LRRK2 in a manner similar to other mutations in this region (eg, pathogenic R1441G/C/H hotspot variants or Y1699C). Based on our functional data and the American College of Medical Genetics and Genomics (ACMG) criteria,^9^ we recommend LRRK2 p.V1447L variant reclassification from VUS to likely pathogenic.

## 1 Methods

Detailed descriptions of all materials and methods can be found in the Supplementary material. These include isolation of peripheral blood neutrophils and monocytes from fresh blood with ex vivo treatment with and without the specific LRRK2 kinase inhibitor MLi‐2 (200 nM, 30 min),^10^, ^11^ genetic testing strategy via targeted next‐generation sequencing (NGS) for PD‐relevant findings, structural mapping of LRRK2 variants within the published high‐resolution cryogenic electron microscopy (cryo‐EM) structure of full‐length inactive LRRK2 (PDB: 7LI4),^12^ HEK293 overexpression of LRRK2 variants, and LRRK2 kinase pathway analysis by quantitative immunoblotting.^13^ The following databases were used for cross‐referencing the occurrence of LRRK2 variants in the general population and in people with PD: MDSGene database (https://www.mdsgene.org),^14^ PDvariant browser (https://pdgenetics.shinyapps.io/VariantBrowser/),^15^ and gnomAD (v4.1.0) (https://gnomad.broadinstitute.org/).^16^

## 2 Results

### 2.1 Index Patient and Genetic Testing

The patient is a Caucasian woman in her early 50s, who was diagnosed with PD aged 42 years. There was no known family history of PD or tremors. Because of the relatively early age at PD onset and more rapid symptom progression with motor impairment at the age of 45 years, along with some atypical clinical features, genetic testing via targeted NGS was performed. This did not reveal a pathogenic variant or relevant copy number variation in any of the common PD‐associated genes; instead, a rare heterozygous LRRK2 VUS (c.4339G>C, p.V1447L) and carrier status for a common GBA1 PD risk variant, p.T408M (c.1223C>T), were identified.

To our knowledge, the LRRK2 p.V1447L variant has not been previously reported in PD patients, nor is it listed in the literature,^8^ including the MDSGene database or the PDvariant browser.^14^, ^15^ It is also absent from gnomAD (v4.1.0).^16^ However, MDSGene reports six PD cases with a methionine substitution at the same residue (p.V1447M), which has previously been shown to increase LRRK2 kinase activity in a robust cellular assay.^13^ This variant is absent from gnomAD. Other substitutions at this site, as reported in gnomAD, include p.V1447E and p.V1447V (each observed once) and p.V1447G (18 heterozygous cases). None of these have been linked to PD patients or functionally characterized.

### 2.2 Elevated LRRK2 Kinase Activity in Peripheral Blood of the LRRK2 p.V1447L Carrier with PD

**FIG. 1:**
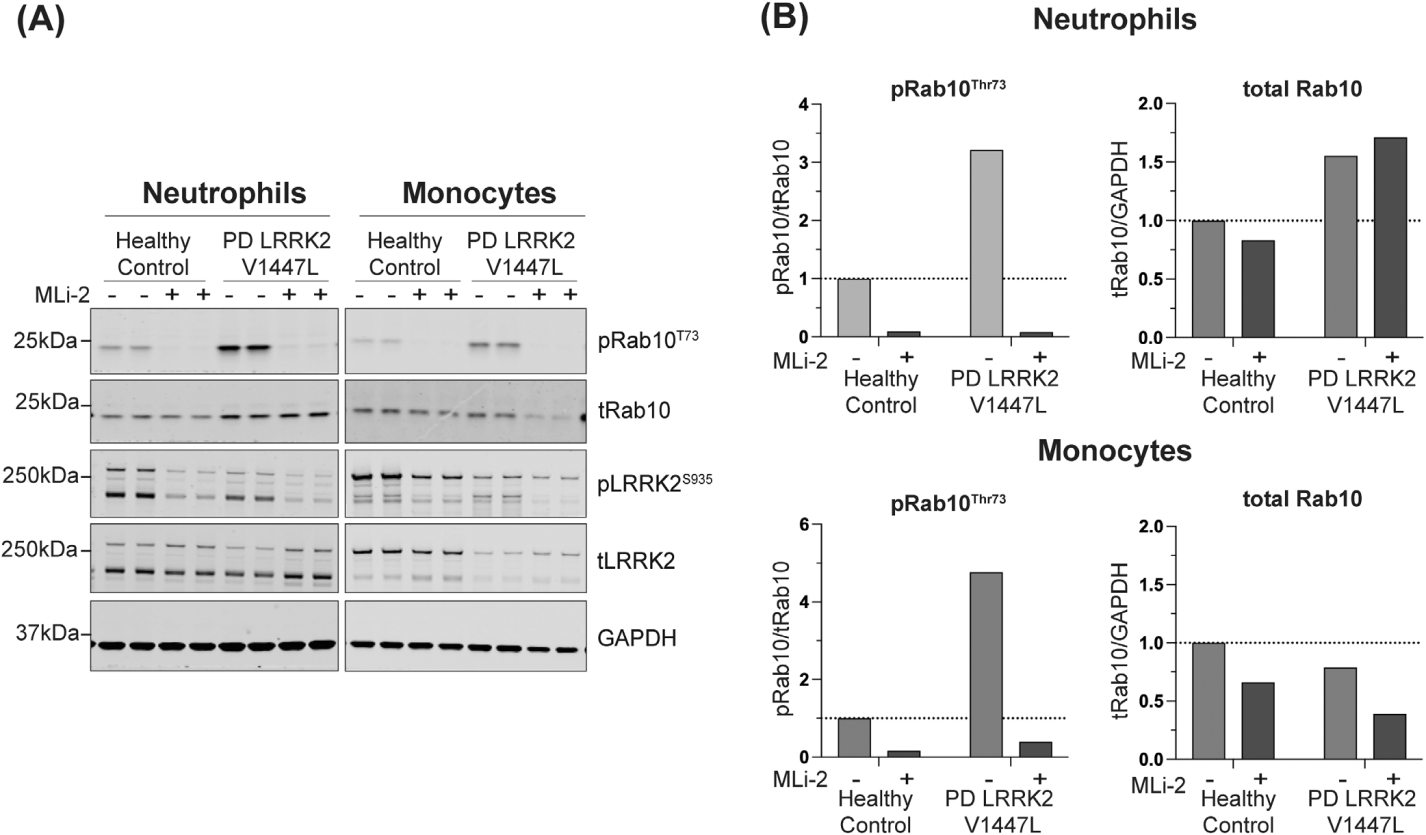
LRRK2 V1447L variant increases LRRK2‐dependent Rab10^Thr73^ phosphorylation in Parkinson's disease (PD) patient‐derived clinical samples. (A) Neutrophils and monocytes were isolated from a heterozygous LRRK2 p.V1447L variant carrier with PD and an age‐matched healthy control. Samples were treated ex vivo with or without 200 nM MLi‐2 for 30 min and 10 μg of each sample was loaded. Following Western blot analysis, membranes were incubated with the indicated antibodies and imaged using a LICOR Odyssey CLx imaging system. Quantified results were normalized to the healthy control and expressed as (B) pRab10/total Rab10 and total Rab10/GAPDH.

We assessed whether the PD patient with the LRRK2 p.V1447L variant showed increased LRRK2 kinase activity in peripheral blood. Using phosphorylation of Rab10 at threonine 73 (pThr73‐Rab10) – a well‐established readout of LRRK2 kinase activity^11^, ^17^ – we found that pThr73‐Rab10 levels were over 3‐fold higher in both peripheral blood neutrophils and monocytes compared with a healthy control (Fig. 1A, B). Treatment with the LRRK2 kinase inhibitor MLi‐2 resulted in near complete dephosphorylation of pThr73‐Rab10, confirming that the observed phosphorylation was LRRK2 kinase‐dependent.

### 2.3 Structural Mapping of the LRRK2 p.V1447L Variant

**FIG. 2:**
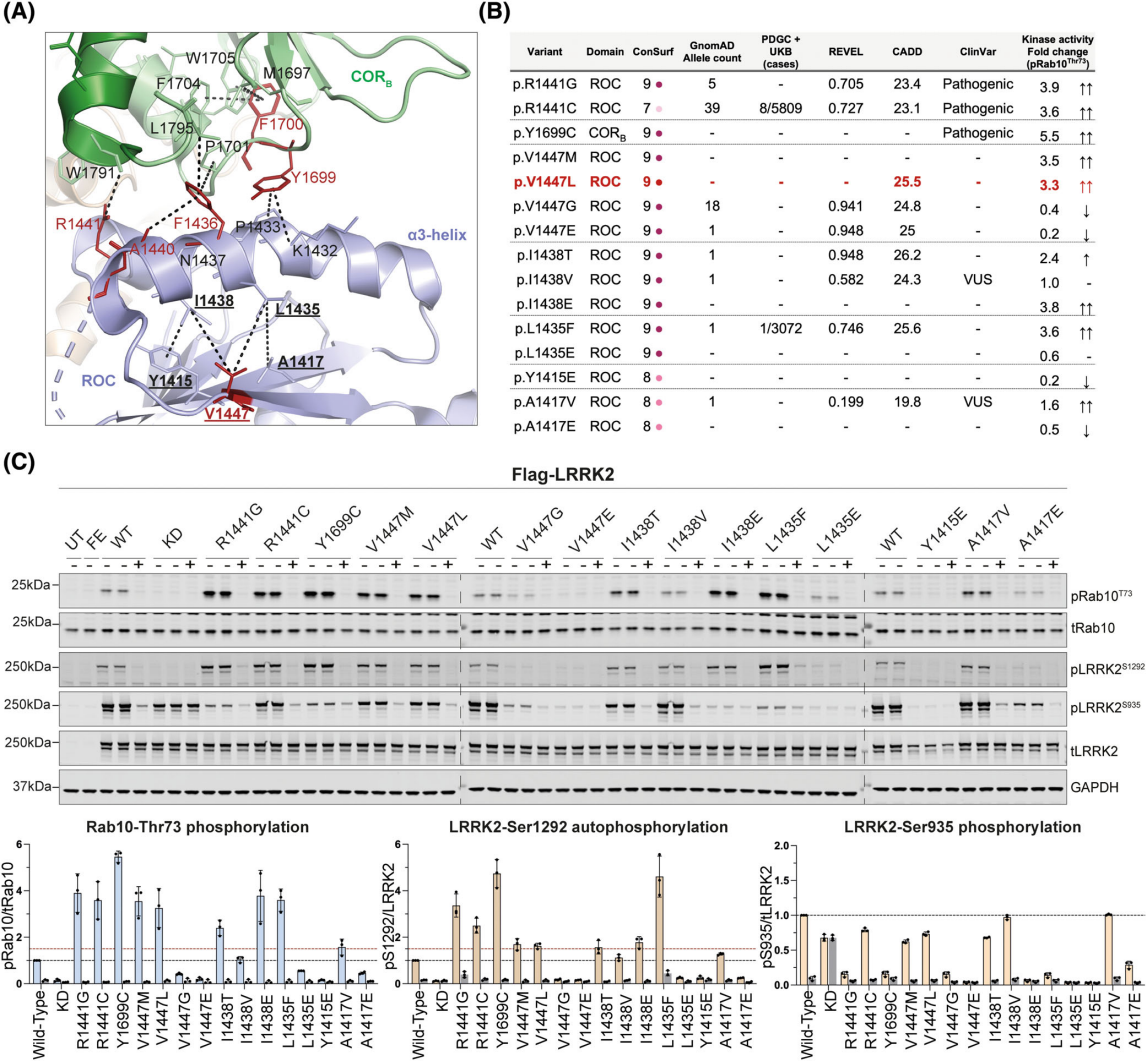
Structural and functional analysis of LRRK2 variants at the ROC:COR_B_ interdomain interface identified several novel kinase‐activating variants. (A) Structural representation of the ROC:COR_B_ interdomain interface, obtained using cryogenic electron microscopy structure of full‐length inactive LRRK2 (PDB, 7LI4).^12^ Indicated in red are residues where pathogenic LRRK2‐activating variants have been identified. (B) Table representing the evolutionary conservation and frequency of LRRK2 variants in the general healthy population and Parkinson's disease (PD) patients. REVEL and CADD scores provide in silico predictions regarding the probability that these variants may be pathogenic. Finally, the table summarizes the findings from the cellular assay of the LRRK2 variant analysis. Fold change >3‐fold indicates that the variant is strongly activating (↑↑) while <3 but >1.5‐fold indicates a moderately activating variant (↑). Conversely, a downward arrow (↓) indicates that pRab10 levels are <0.5 relative to LRRK2 wild‐type and therefore inactivating. The quantified results are representative of the average of three biological replicates. (C) Functional analysis of new variants within the ROC:COR_B_ interdomain interface using an overexpression assay in HEK293 cells with wild‐type LRRK2 (WT) and kinase dead (KD=D2017A) as positive and negative controls, respectively. Cell lysates (20 μg) were subjected to multiplexed immunoblot analysis, and the quantified results were then normalized to the LRRK2 wild‐type and expressed as pRab10/total Rab10, pSer935/total LRRK2, and pSer1292/total LRRK2. The red dotted line at y = 1.5 emphasizes phosphorylation levels above 1.5‐fold relative to the LRRK2 wild‐type. Each datapoint represents one biological replicate.

Analysis of the high‐resolution cryo‐EM structure of full‐length inactive LRRK2 (PDB, 7LI4)^12^ reveals that the V1447 residue is located within the ROC domain, within a beta‐sheet region that interacts with the α3‐helix (residues 1424–1442) on the ROC surface^18^ (Fig. 2A). V1447 is highly conserved across species (Consurf score of 9/9),^19^ suggesting a functionally important role. Notably, the α3‐helix harbors known pathogenic variants (p.A1440P^20^ and p.R1441G^21^/C^4^/H^21^, ^22^) that activate the LRRK2 kinase (Fig. 2A). The surface of the α3‐helix opposite to V1447 interacts with the COR_B_ domain (Fig. 2A), which includes additional activating LRRK2 variants such as p.Y1699C^4^ and p.F1700L.^23^ This suggests that the network of interactions between the ROC beta‐sheet, α3‐helix, and COR_B_ is critical in stabilizing the inactive form of LRRK2. Previous work showed that disrupting the ROC‐COR_B_ promotes LRRK2 activation.^12^, ^13^, ^24^ Our hypothesis is that the p.V1447L variant in the beta‐sheet region, similarly to p.V1447M,^13^ impacts the positioning of the α3‐helix, thereby destabilizing the ROC‐COR_B_ interface and shifting LRRK2 towards its active conformation (Fig. 2A).

### 2.4 Functional Analysis of Variants At and Around the LRRK2 V1447 and ROC:COR_B_ Interface

To further test our hypothesis, we examined how mutations at and around the LRRK2 V1447 residue affect kinase activity, focusing on residues at the beta‐sheet:α3‐helix interface (Fig. 2A). We selected five highly conserved residues, namely Y1415 and A1417 (beta‐sheet interface), L1435 and I1438 (α3‐helix interface), and V1447 (α3‐helix). Reviewing PD databases^14^, ^15^ and gnomAD,^16^ we identified six naturally occurring variants at these sites including p.V1447G, p.V1447E, p.I1438T, p.I1438V, p.L1435F, and p.A1417V (Fig. 2B). We assessed their functional effect on LRRK2 kinase activity using our robust HEK293 overexpression assay,^13^ alongside mutations not reported in the literature or gnomAD including p.I1438E, p.L1435E, p.Y1415E, and p.A1417E (Fig. 2B).

The novel p.V1447L variant identified in our PD patient, along with the previously reported p.V1447M, strongly activated LRRK2 kinase, increasing Rab10 substrate phosphorylation 3.5‐fold and moderately enhancing LRRK2 autophosphorylation at Ser1292 (1.5‐fold) relative to LRRK2 wild‐type (Fig. 2C, Tables S1, S2). LRRK2 phosphorylation at Ser935 was slightly reduced. In contrast, p.V1447G and p.V1447E reduced Rab10 phosphorylation and abolished both Ser1292 and Ser935 LRRK2 phosphorylation (Fig. 2C, Tables S1, S2).

At residue I1438, substitutions to glutamic acid (p.I1438E) and threonine (p.I1438T) increased Rab10 phosphorylation 2‐ and 4‐fold, respectively, with both enhancing Ser1292 autophosphorylation, while only p.I1438T reduced Ser935 phosphorylation. The I1438V variant had no detectable effect. All three variants are rare, with p.I1438E not present in the any of the databases that we interrogated and p.I1438T and p.I1438V being present once each in heterozygous European carriers in gnomAD (Fig. 2B, C, Tables S1, S2).

The nearby L1435F variant, located within the α3‐helix, was identified in a PD patient^15^ and once in gnomAD (heterozygous South Asian carrier) and increased LRRK2‐dependent Rab10 phosphorylation ~4‐fold. Substitutions to glutamic acid at this residue (p.L1435E), not reported in humans, reduced LRRK2 kinase activity. Similarly, p.A1417E, not reported in humans, decreased LRRK2 activity, while the rare p.A1417V variant (seen once in a heterozygous European carrier) mildly increased Rab 10 phosphorylation (1.5‐fold) without affecting Ser1292 or Ser935 phosphorylation (Fig. 2B, C, Tables S1, S2).

## 3 Discussion

We describe a sporadic PD patient with onset in her early 40s, carrying a rare heterozygous LRRK2 p.V1447L variant of unknown clinical significance, along with a GBA1 p.T408M risk variant. While the GBA1 variant may contribute to the earlier disease onset,^25^ we focused on the functional impact of the LRRK2 p.V1447L variant. We observed marked LRRK2 kinase pathway activation, with Rab10 substrate phosphorylation as a readout, in patient‐derived peripheral blood neutrophils and monocytes compared with an unrelated healthy control (Fig. 1). This was confirmed by an over 3‐fold increase in LRRK2 kinase activity in HEK293 cells overexpressing the LRRK2 V1447L variant. This activation matches the effect size of pathogenic LRRK2 R1441 hotspot variants (Fig. 2C) and exceeds the common p.G2019S variant (around 1.5‐fold).^13^, ^26^

Previous studies^12^ have shown that the ROC:COR_B_ interface is a hotspot for some of the most activating pathogenic LRRK2 variants (eg, p. A1440P,^20^ p.R1441G^21^/C^4^/H^21^, ^22^ or p.Y1699C^4^, and p.F1700L^23^). Although V1447 does not directly map into the ROC:COR_B_ interface, it lies within a hydrophobic region that interacts and likely stabilizes the α3‐helix interaction with the COR_B_ interface, maintaining LRRK2's inactive conformation. Notably, the V1447 residue lies within a region of the ROC beta‐sheet that connects the α3‐helix and the COR_B_ domain. This suggests that V1447 plays a role in maintaining the confirmational integrity of this regulatory interface. Mutations at I1438, an adjacent α3‐helix residue interacting with this region, also activate LRRK2 when replaced with glutamic acid or threonine. Conversely, substitutions such as p.Y1415E, p.A1417E, p.V1447G, and p.V1447E reduced kinase activity, possibly by reinforcing the inactive state or disrupting catalytic function. These findings support the hypothesis that substitutions in this critical structural network modulate kinase activity by affecting LRRK2 conformational stability.

Our results highlight the importance of performing functional studies while also considering structural implications when evaluating the pathogenicity of LRRK2 missense variants, including different substitutions at the same residue. Demonstrating increased kinase activity in cell‐based assays, particularly when corroborated in vivo using human blood samples, provides strong evidence that a variant exerts its pathogenic effects via the established mechanism of LRRK2 kinase activation.^6^, ^23^ In contrast, at least in the heterozygous state, variants that exhibit reduced kinase activity are unlikely to be pathogenic as individuals with heterozygous loss‐of‐function LRRK2 variants are generally healthy.^27^ Interestingly, our findings also expand the spectrum of potential loss‐of‐kinase function variants in LRRK2 to include missense variants. As genome sequencing becomes more widely integrated into clinical care, and with the advent of LRRK2‐targeted therapies,^3^ there is an urgent need to systematically annotate variants of unknown clinical significance.

Specifically, we clearly demonstrate that the rare LRRK2 p.V1447L variant increases kinase activity, consistent with the known pathogenic mechanism of LRRK2 mutations in PD. In line with ACMG guidelines,^9^ we recommend reclassifying this variant from a variant of uncertain significance to likely pathogenic, and suggest that individuals carrying this variant be considered for inclusion in LRRK2‐targeted treatment approaches.

## Author Roles

(1) Research Project: A. Conception B. Organization C. Execution; (2) Statistical Analysis: A. Design B. Execution C. Review and Critique; (3) Manuscript Preparation: A. Writing of the First Draft B. Review and Critique.

N.P.: 1A, 1B, 1C, 2A, 2B, 3A.

P.L.: 1A, 1C, 3A, 3B.

S.W.: 2C, 3B.

N.G.: 2C, 3B.

L.A.: 1A, 2C, 3B.

D.R.A.: 1A, 3B.

E.S.: 1A, 1B, 2A, 3A.

## Supporting information


**Data S1.** Supporting Information.

## Data Availability

Data supporting the findings of this study can be found in Supporting Information including the unedited Western blots and quantifications thereof.
