# Supplementary material for: Functional and Structural Characterization of LRRK2 p.V1447L in Parkinson's Disease
Source: Mov Disord. 2025 Jul 30;40(10):2251–6. doi: 10.1002/mds.30284 (PMC12553989; doi:10.1002/mds.30284)
Supplement: Supplementary file 1 — Data S1. Supporting Information. [file MDS-40-2251-s001.docx]

**Supplementary Table 1.**

**Supplementary Table 1. pSer1292/LRRK2 levels across all LRRK2 variants expressed in HEK293 cells.** Quantification of phosphorylated LRRK2 at Ser1292 relative to total LRRK2 in wild-type and mutant LRRK2, treated with DMSO or MLi-2, with three biological replicates per condition. The raw values were normalized to LRRK2 wild-type and expressed as fold changes relative to the LRRK2 wild-type protein.

|  |  | pSer1292/LRRK2 | | | | | |  |
| --- | --- | --- | --- | --- | --- | --- | --- | --- |
|  | *DMSO* | | |  | *MLi-2* | | |  |
|  | **Replicate 1** | **Replicate 2** | **Replicate 3** | **Average** | **Replicate 1** | **Replicate 2** | **Replicate 3** | **Average** |
| Wild-Type | 1.00 | 1.00 | 1.00 | 1.00 | 0.14 | 0.16 | 0.17 | 0.16 |
| KD | 0.10 | 0.11 | 0.13 | 0.11 | 0.10 | 0.12 | 0.16 | 0.13 |
| R1441G | 3.05 | 3.95 | 3.12 | 3.37 | 0.27 | 0.53 | 0.38 | 0.39 |
| R1441C | 2.22 | 2.85 | 2.43 | 2.50 | 0.14 | 0.20 | 0.22 | 0.19 |
| Y1699C | 4.12 | 5.33 | 4.78 | 4.74 | 0.13 | 0.15 | 0.20 | 0.16 |
| V1447M | 1.74 | 1.92 | 1.43 | 1.70 | 0.10 | 0.07 | 0.17 | 0.11 |
| V1447L | 1.66 | 1.72 | 1.49 | 1.63 | 0.11 | 0.11 | 0.18 | 0.13 |
| V1447G | 0.17 | 0.21 | 0.15 | 0.18 | 0.06 | 0.13 | 0.10 | 0.10 |
| V1447E | 0.15 | 0.17 | 0.16 | 0.16 | 0.07 | 0.12 | 0.09 | 0.10 |
| I1438T | 1.49 | 1.89 | 1.32 | 1.57 | 0.08 | 0.12 | 0.08 | 0.10 |
| I1438V | 1.12 | 1.26 | 0.99 | 1.12 | 0.12 | 0.17 | 0.14 | 0.15 |
| I1438E | 1.92 | 1.93 | 1.50 | 1.78 | 0.07 | 0.11 | 0.15 | 0.11 |
| L1435F | 4.45 | 5.56 | 3.82 | 4.61 | 0.37 | 0.57 | 0.40 | 0.45 |
| L1435E | 0.24 | 0.23 | 0.29 | 0.25 | 0.05 | 0.10 | 0.13 | 0.10 |
| Y1415E | 0.21 | 0.23 | 0.34 | 0.26 | 0.16 | 0.12 | 0.22 | 0.17 |
| A1417V | 1.23 | 1.30 | 1.31 | 1.28 | 0.11 | 0.14 | 0.18 | 0.14 |
| A1417E | 0.24 | 0.26 | 0.25 | 0.25 | 0.05 | 0.10 | 0.03 | 0.06 |

**Supplementary Table 2.**

**Supplementary Table 2. pSer935/LRRK2 levels across all LRRK2 variants expressed in HEK293 cells.** Quantification of phosphorylated LRRK2 at Ser935 relative to total LRRK2 in wild-type and mutant LRRK2, treated with DMSO or MLi-2, with three biological replicates per condition. The values were normalized to LRRK2 wild-type and expressed as fold changes in respect to the LRRK2 wild-type protein.

|  |  | pSer935/LRRK2 | | | | | |  |
| --- | --- | --- | --- | --- | --- | --- | --- | --- |
|  | *DMSO* | | |  | *MLi-2* | | |  |
|  | **Replicate 1** | **Replicate 2** | **Replicate 3** | **Average** | **Replicate 1** | **Replicate 2** | **Replicate 3** | **Average** |
| Wild-Type | 1.00 | 1.00 | 1.00 | 1.00 | 0.12 | 0.10 | 0.06 | 0.09 |
| KD | 0.73 | 0.66 | 0.64 | 0.68 | 0.72 | 0.67 | 0.64 | 0.68 |
| R1441G | 0.19 | 0.17 | 0.11 | 0.16 | 0.06 | 0.07 | 0.04 | 0.06 |
| R1441C | 0.77 | 0.78 | 0.82 | 0.79 | 0.08 | 0.07 | 0.06 | 0.07 |
| Y1699C | 0.19 | 0.18 | 0.12 | 0.16 | 0.09 | 0.11 | 0.06 | 0.09 |
| V1447M | 0.62 | 0.65 | 0.61 | 0.62 | 0.07 | 0.06 | 0.04 | 0.06 |
| V1447L | 0.73 | 0.76 | 0.71 | 0.73 | 0.07 | 0.07 | 0.05 | 0.06 |
| V1447G | 0.18 | 0.17 | 0.11 | 0.15 | 0.05 | 0.05 | 0.03 | 0.04 |
| V1447E | 0.05 | 0.05 | 0.02 | 0.04 | 0.04 | 0.04 | 0.02 | 0.03 |
| I1438T | 0.69 | 0.68 | 0.67 | 0.68 | 0.06 | 0.07 | 0.04 | 0.06 |
| I1438V | 1.00 | 0.94 | 0.98 | 0.97 | 0.08 | 0.10 | 0.06 | 0.08 |
| I1438E | 0.07 | 0.07 | 0.05 | 0.06 | 0.06 | 0.06 | 0.03 | 0.05 |
| L1435F | 0.17 | 0.16 | 0.10 | 0.14 | 0.08 | 0.07 | 0.04 | 0.06 |
| L1435E | 0.04 | 0.04 | 0.02 | 0.04 | 0.04 | 0.04 | 0.02 | 0.03 |
| Y1415E | 0.04 | 0.04 | 0.02 | 0.03 | 0.04 | 0.03 | 0.02 | 0.03 |
| A1417V | 1.00 | 1.01 | 1.02 | 1.01 | 0.10 | 0.11 | 0.06 | 0.09 |
| A1417E | 0.32 | 0.32 | 0.23 | 0.29 | 0.04 | 0.04 | 0.02 | 0.03 |

**Materials and Methods**

**Peripheral blood collection and neutrophil & monocyte isolation**

For the analysis of LRRK2 kinase pathway activity in human samples, 40ml of fresh blood was collected via venesection from the patient and an unrelated aged-matched healthy control for immediate peripheral blood neutrophil and monocyte isolations via immunomagnetic negative selection, consistent with previous methods^1,2^. Prior to lysis, the cells were treated *ex vivo* with or without the specific LRRK2 kinase inhibitor MLi-2 (200nM, 30min), synthesized by Natalia Shpiro (University of Dundee).

**Genetic analysis**

DNA extraction was from whole blood for targeted next-generation sequencing (NGS) panel comprising 14 genes associated with Parkinson’s Disease (*ATP1A3, ATP13A2, DNAJC6, FBXO7, GCH1, LRRK2, PARK2, PARK7, PINK1, PLA2G6, SNCA, SYNJ1, VPS13C and VPS35*) was performed using Illumina NextSeq. Coding regions and flanking intronic regions were captured using KAPA HyperCap system (Roche). Sequencing quality criteria included a minimum read depth of ≥30X for at least 99% of the targeted bases, ensuring a detection sensitivity of at least 99% for single nucleotide variants. The GBA1 variant was detected using another targeted NGS panel using Illumina NextSeq system that covered coding regions and flanking regions of 84 lysosomal genes which were captured by solution hybridization (SureSelect XT HS, Agilent). Sequencing quality criteria included a minimum of ≥30X reads for at least 99% of the targeted bases.

**Plasmids, cell culture and transient transfection in HEK293 cells**

All plasmids are available from the MRC PPU Reagents and Services (<https://mrcppureagents.dundee.ac.uk>) (see Supplementary Table 3). HEK293 overexpression assay was performed as described previously^3^. Briefly, HEK293 cells were grown in DMEM (Dulbecco s Modified Eagle Medium) supplemented with 10% (v/v) foetal calf serum (FBS), 2 mM L-glutamine, 100 U/ml penicillin. The cells were seeded in 6-well plates and at 80-90% confluency transfected with 2ug of each of the Flag-LRRK2 plasmids, including Flag-Empty control plasmid using polyethylenimine (PEI) transfection reagent (1:3 DNA:PEI ratio) all in 300uL Opti-MEM (Gibco). 16-20 hours after transfections, the cells were treated with either DMSO or LRRK2 kinase inhibitor MLi-2 (200nM, 90min) and lysed with ice cold 1% (v/v) Triton lysis buffer. The cell debris was cleared by centrifuging the samples at 17 000g for 15min at 4°C and protein concentration determined by BCA protein assay (Thermo Fisher Scientific Cat #23225).

Supplementary Table 3: Plasmids generated and used in this study:

| DU Number | Construct | Plasmid | TRANSCRIPT ID |
| --- | --- | --- | --- |
| DU41799 | Flag Empty | pCMV5 | - |
| DU6841 | Flag LRRK2 Wild-type | pCMV5 | ENST00000298910.12 |
| DU80014 | Flag LRRK2 Y1415E | pCMV5 | ENST00000298910.12 |
| DU77993 | Flag LRRK2 A1417V | pCMV5 | ENST00000298910.12 |
| DU77994 | Flag LRRK2 A1417E | pCMV5 | ENST00000298910.12 |
| DU77991 | Flag LRRK2 L1435F | pCMV5 | ENST00000298910.12 |
| DU77992 | Flag LRRK2 L1435E | pCMV5 | ENST00000298910.12 |
| DU77990 | Flag LRRK2 I1438T | pCMV5 | ENST00000298910.12 |
| DU80013 | Flag LRRK2 I1438V | pCMV5 | ENST00000298910.12 |
| DU77995 | Flag LRRK2 I1438E | pCMV5 | ENST00000298910.12 |
| DU26477 | Flag LRRK2 R1441G | pCMV5 | ENST00000298910.12 |
| DU13078 | Flag LRRK2 R1441C | pCMV5 | ENST00000298910.12 |
| DU62501 | Flag LRRK2 V1447M | pCMV5 | ENST00000298910.12 |
| DU77444 | Flag LRRK2 V1447L | pCMV5 | ENST00000298910.12 |
| DU77977 | Flag LRRK2 V1447G | pCMV5 | ENST00000298910.12 |
| DU77981 | Flag LRRK2 V1447E | pCMV5 | ENST00000298910.12 |
| DU26486 | Flag LRRK2 Y1699C | pCMV5 | ENST00000298910.12 |
| DU10128 | Flag LRRK2 D2017A (Kinase dead) | pCMV5 | ENST00000298910.12 |

**Quantitative immunoblot analysis**

Cell lysates were prepared at a concentration of 2µg/µL in NuPage LDS Sample Buffer (x4) with 5% β-mercaptoethanol and boiled at 96°C for 10min. 10ug or 20ug of each sample was loaded on the NuPAGE Bis-Tris 4-12% gradient gels and electrophorized at 100V for ~2h. Using a nitrocellulose membrane, the gels were transferred at 90V for 90min in 1X Transfer Buffer (48mM Tris-HCl and 39mM glycine) and blocked for 30min in 5% skim dry milk diluted in TBS-T. The membranes were left to incubate overnight at 4°C with primary antibodies diluted at 1 µg/ml: multiplexed monoclonal anti-LRRK2 mouse (NeuroMab #75-253) and anti-pS935 LRRK2 rabbit (Abcam #ab133450) and multiplexed monoclonal anti-Rab10 mouse (Nanotools #0680-100) and anti-MJFF-pRab10 rabbit (Abcam #ab230261) antibodies. LRRK2 autophosphorylation site was visualized using monoclonal anti-pS1292 LRRK2 rabbit (Abcam #ab203181) at 1:1000 dilution. GAPDH (Santa Cruz #sc-32233) was used as a loading control at 1:5000. After 30min wash, the membranes were incubated 1h at room-temperature with multiplexed fluorescent secondary antibodies: 1:10000 goat anti-mouse IRDye 680LT and 1:10000 goat anti-rabbit IRDye 800CW (LI-COR). Finally, the membranes were rinsed, and the signals acquired with LI-COR Odyssey CLx imaging system. Post data analysis the results were visualized using GraphPad Prism V10.0.3.

**Structural mapping of LRRK2 variants**

Structural mapping was performed using an existing high resolution Cryo-EM structure of full length inactive LRRK2 (PDB, 7LI4) with structural visualization software, PyMOL 3.0.

**References**

1. Fan Y, Howden AJ, Sarhan AR, Lis P, Ito G, Martinez TN, et al. Interrogating Parkinson's disease LRRK2 kinase pathway activity by assessing Rab10 phosphorylation in human neutrophils. Biochemical Journal. 2018;475(1):23-44.

2. Mir R, Tonelli F, Lis P, Macartney T, Polinski NK, Martinez TN, et al. The Parkinson's disease VPS35[D620N] mutation enhances LRRK2-mediated Rab protein phosphorylation in mouse and human. Biochem J. 2018 Jun 6;475(11):1861-1883.

3. Kalogeropulou AF, Purlyte E, Tonelli F, Lange SM, Wightman M, Prescott AR, et al. Impact of 100 LRRK2 variants linked to Parkinson's disease on kinase activity and microtubule binding. Biochem J. 2022;479(17):1759-83.

**Supplementary data – Unedited Western Blots**

**Raw Western Blot files**


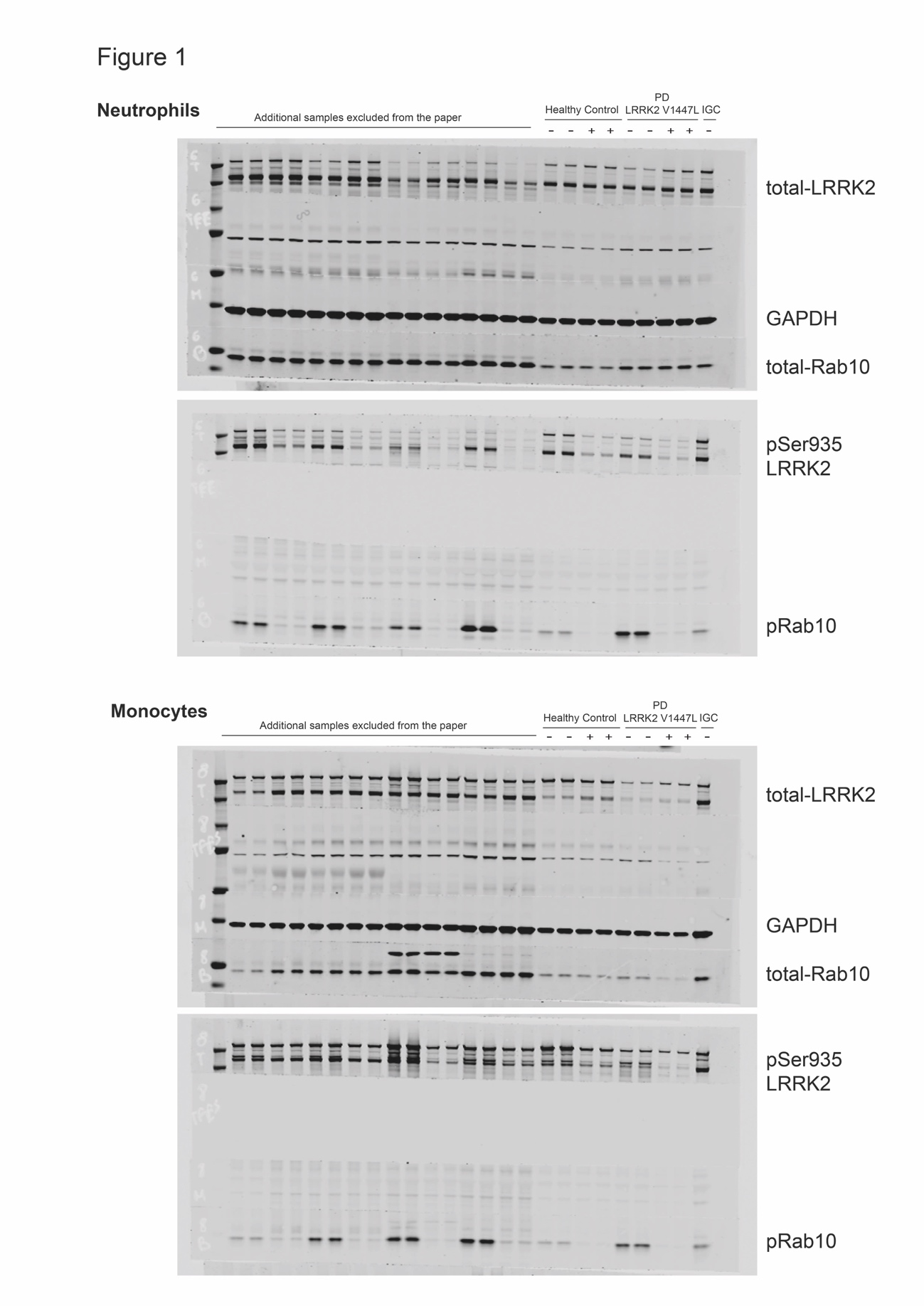
Comprehensive Western Blot files visualizing the raw images as acquired from the LICOR Odyssey CLx. The images are provided for each figure, along with additional biological replicates associated with Figure 2C that are not included in the primary manuscript.


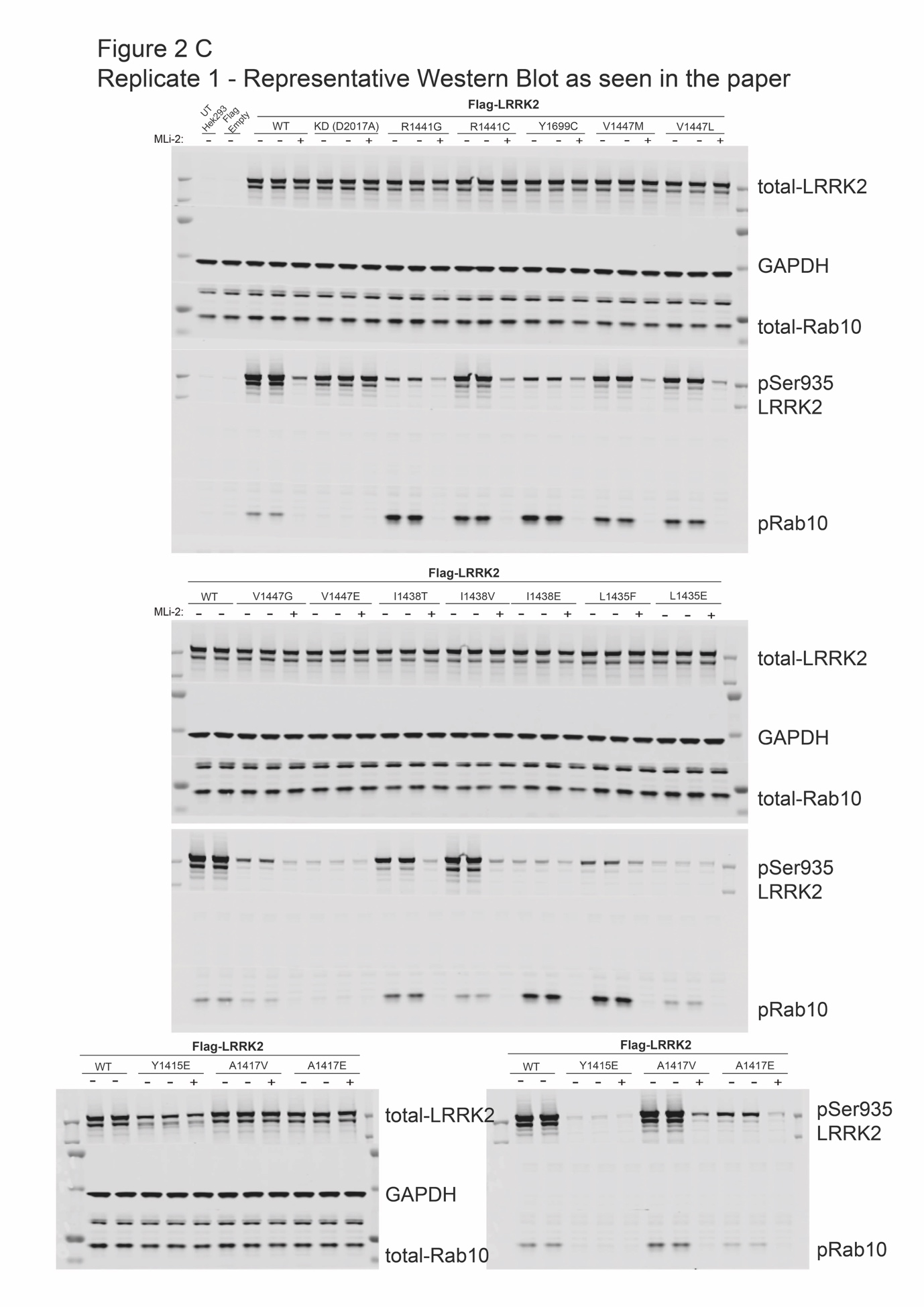


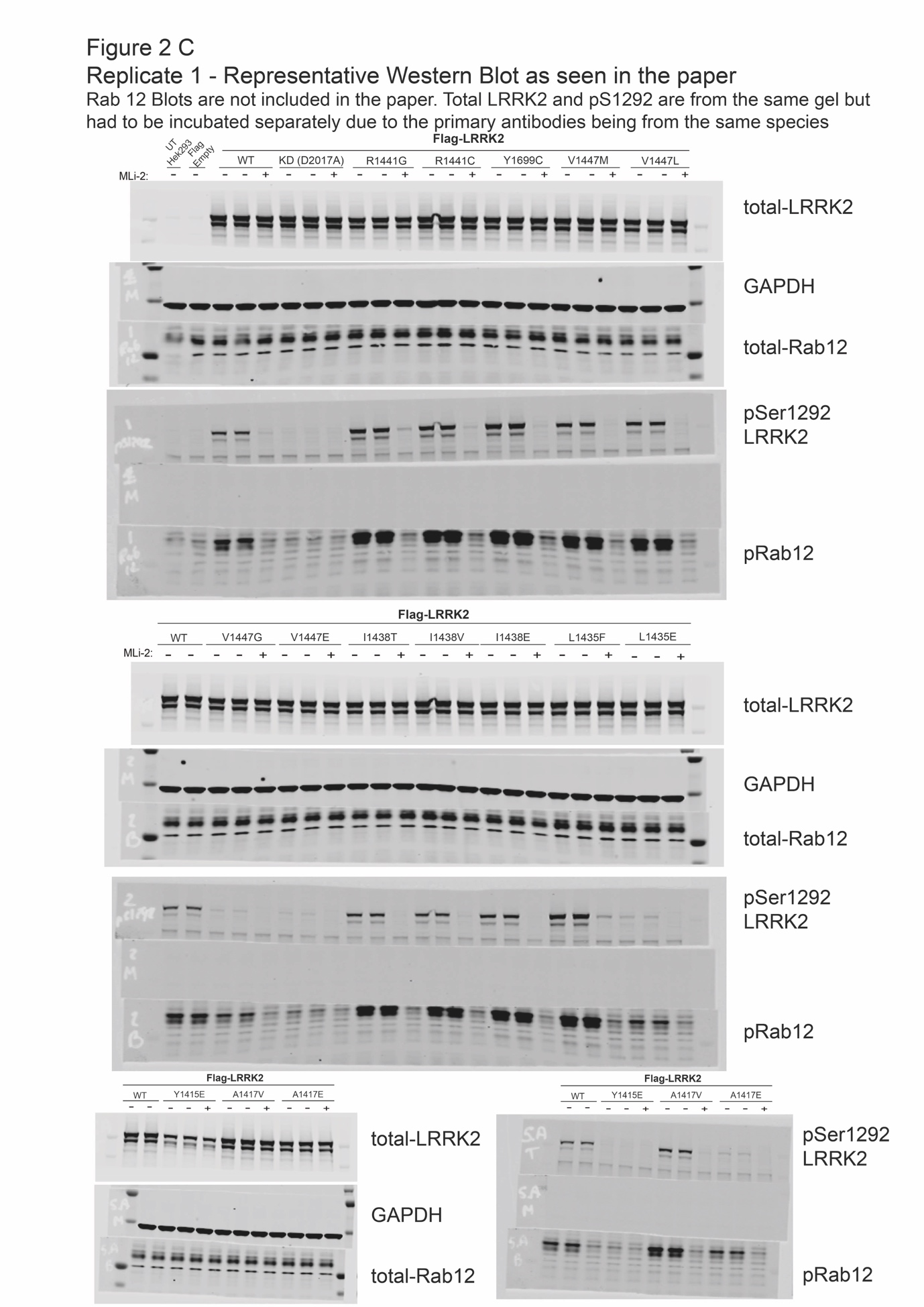


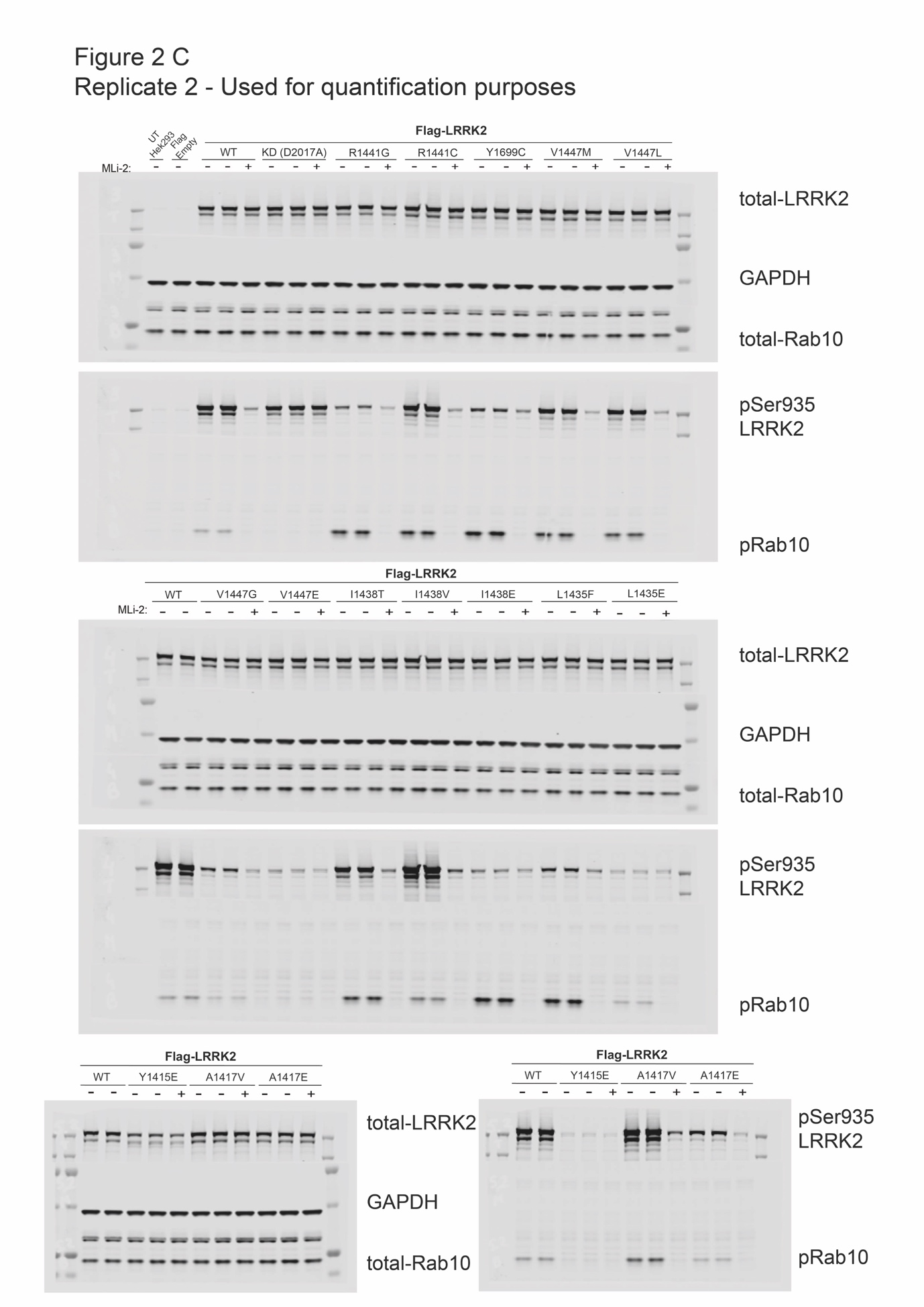

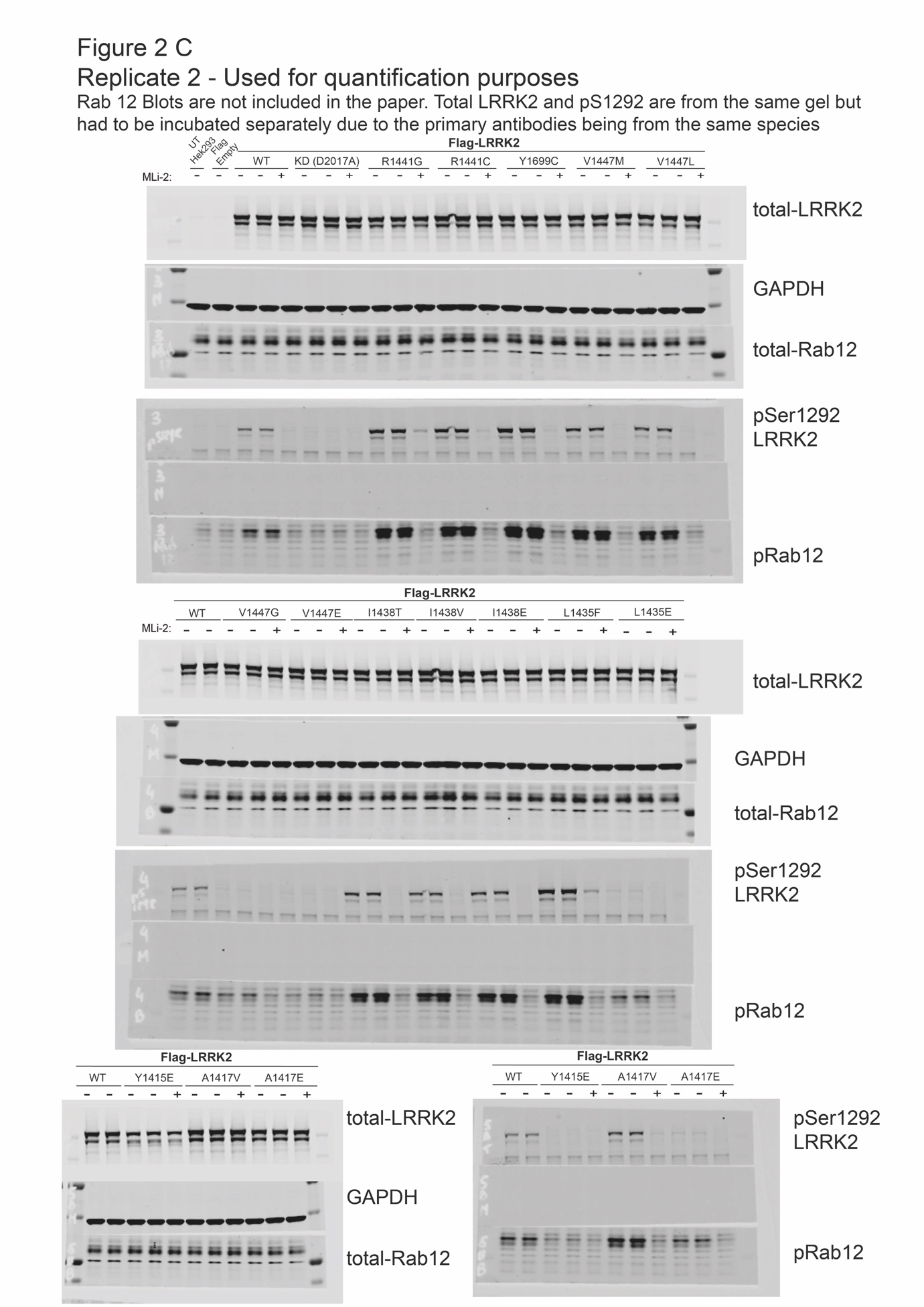

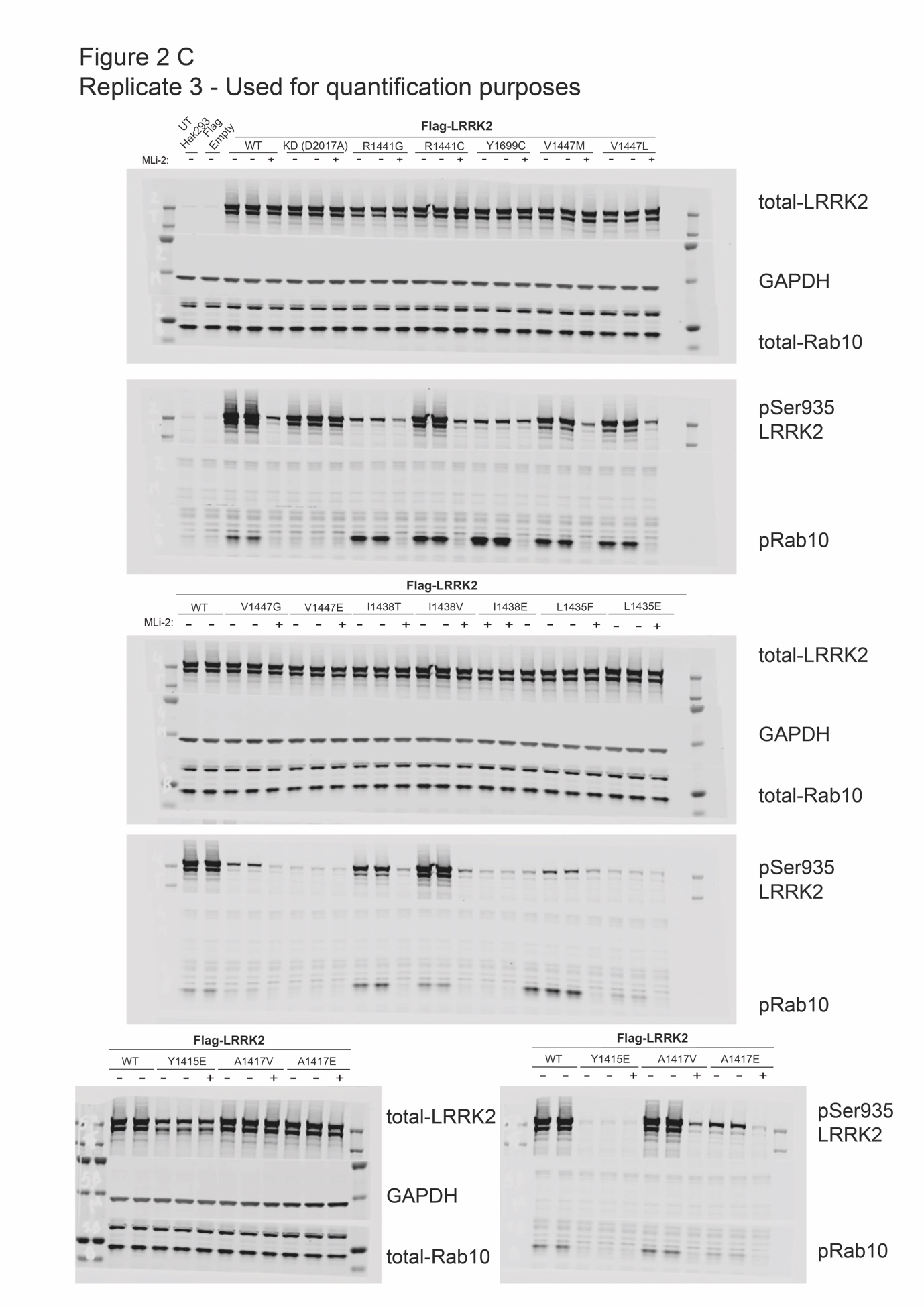

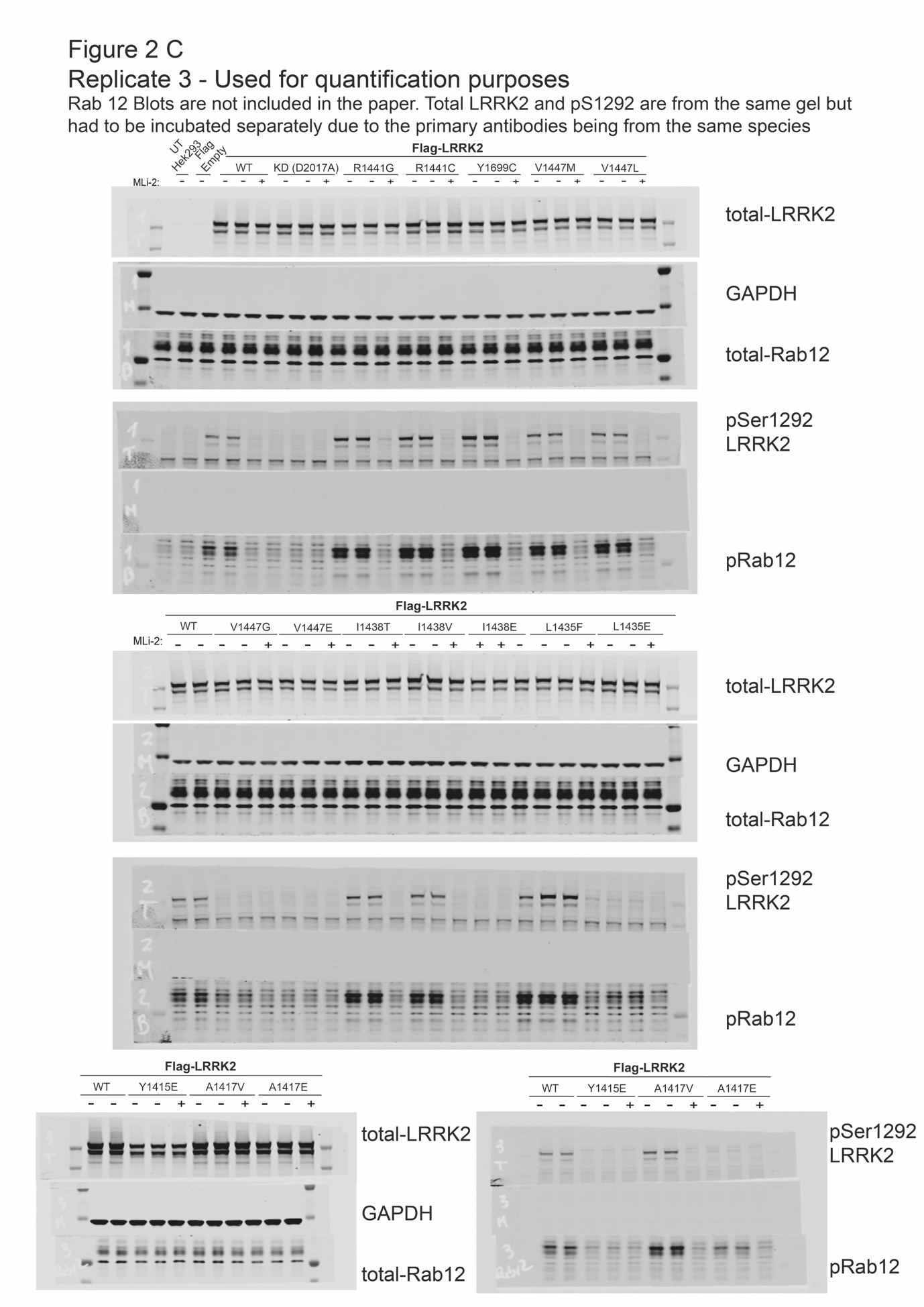


**Supplementary data – Quantified and analysed values from immunoblotting assay as illustrated in**

**Figure 1B**

Quantification of pRab10 normalized to total Rab10 and total Rab10 normalized to GAPDH in neutrophils and monocytes from a healthy control and LRRK2 V1447L mutation carrier, treated with DMSO or MLi-2.

**Neutrophils**

| pRab10/tRab10 | | |  | tRab10/GAPDH | | |
| --- | --- | --- | --- | --- | --- | --- |
|  | *DMSO* | *MLi-2* |  |  | *DMSO* | *MLi-2* |
| Healthy control | 1 | 0.094 |  | **Healthy control** | 1 | 0.832 |
| LRRK2 V1447L | 3.214 | 0.081 |  | **LRRK2 V1447L** | 1.553 | 1.711 |

**Monocytes**

| pRab10/tRab10 | | |  | tRab10/GAPDH | | |
| --- | --- | --- | --- | --- | --- | --- |
|  | *DMSO* | *MLi-2* |  |  | *DMSO* | *MLi-2* |
| Healthy control | 1 | 0.163 |  | **Healthy control** | 1 | 0.662 |
| LRRK2 V1447L | 4.77 | 0.39 |  | **LRRK2 V1447L** | 0.789 | 0.39 |

**Supplementary data – Quantified and analysed values from immunoblotting assay as illustrated in Figure 2C**

**pRab10/tRab10** Quantification Across LRRK2 Mutants

Quantification of pRab10 normalized to total Rab10 in wild-type and various LRRK2 mutant under DMSO and MLi-2 treatment, with three biological replicates per condition.

|  | pRab10/tRab10 | | | | | |
| --- | --- | --- | --- | --- | --- | --- |
|  | *DMSO* | | | *MLi-2* | | |
|  | **Replicate 1** | **Replicate 2** | **Replicate 3** | **Replicate 1** | **Replicate 2** | **Replicate 3** |
| Wild-Type | 1 | 1 | 1 | 0.096 | 0.143 | 0.177 |
| KD | 0.097 | 0.14 | 0.204 | 0.059 | 0.077 | 0.064 |
| R1441G | 4.019 | 4.669 | 3.022 | 0.073 | 0.088 | 0.026 |
| R1441C | 3.624 | 4.387 | 2.768 | 0.083 | 0.074 | 0.062 |
| Y1699C | 5.581 | 5.624 | 5.184 | 0.072 | 0.101 | 0.103 |
| V1447M | 3.902 | 3.916 | 2.818 | 0.073 | 0.062 | 0.099 |
| V1447L | 3.358 | 4.039 | 2.369 | 0.093 | 0.115 | 0.169 |
| V1447G | 0.401 | 0.394 | 0.467 | 0.079 | 0.105 | 0.237 |
| V1447E | 0.137 | 0.192 | 0.273 | 0.045 | 0.04 | 0.185 |
| I1438T | 2.449 | 2.711 | 2.051 | 0.059 | 0.075 | 0.185 |
| I1438V | 1.107 | 1.122 | 0.89 | 0.059 | 0.063 | 0.19 |
| I1438E | 4.019 | 4.745 | 2.58 | 0.038 | 0.046 | 0.144 |
| L1435F | 3.722 | 4.003 | 3.065 | 0.072 | 0.06 | 0.178 |
| L1435E | 0.551 | 0.553 | 0.558 | 0.058 | 0.066 | 0.146 |
| Y1415E | 0.127 | 0.153 | 0.179 | 0.072 | 0.04 | 0.101 |
| A1417V | 1.858 | 1.68 | 1.198 | 0.103 | 0.077 | 0.148 |
| A1417E | 0.51 | 0.415 | 0.441 | 0.093 | 0.109 | 0.114 |
